# Risk of introduction of lumpy skin disease in France by the import of vectors in animal trucks

**DOI:** 10.1371/journal.pone.0198506

**Published:** 2018-06-11

**Authors:** Claude Saegerman, Stéphane Bertagnoli, Gilles Meyer, Jean-Pierre Ganière, Philippe Caufour, Kris De Clercq, Philippe Jacquiet, Guillaume Fournié, Claire Hautefeuille, Florence Etore, Jordi Casal

**Affiliations:** 1 Centre of Fundamental and Applied Research for Animals and Health (FARAH), University of Liège, Liège, Belgium; 2 Members of the Expert Committee for Animal Health and Welfare, ANSES, Maisons-Alfort, France; 3 IHAP, University of Toulouse, INRA, ENVT, Toulouse, France; 4 ONIRIS, Nantes, France; 5 CIRAD-INRA ASTRE Joint Research Unit (UMR), BIOS Department, CIRAD, Montpellier, France; 6 CODA-CERVA, Brussels, Belgium; 7 Veterinary Epidemiology, Economics and Public Health Group, Department of Pathobiology and Population Sciences, Royal Veterinary College, Hatfield, United Kingdom; 8 French Agency for Food, Environmental and Occupational Health & Safety (ANSES), Maisons-Alfort, France; 9 Departament de Sanitat I Anatomia Animals. Universitat Autònoma de Barcelona / IRTA-CReSA, Barcelona, Spain; University of Liverpool, UNITED KINGDOM

## Abstract

**Background:**

The lumpy skin disease virus (LSDV) is a dsDNA virus belonging to the *Poxviridae* family and the *Capripoxvirus* genus. Lumpy skin diseases (LSD) is a highly contagious transboundary disease in cattle producing major economic losses. In 2014, the disease was first reported in the European Union (in Cyprus); it was then reported in 2015 (in Greece) and has spread through different Balkan countries in 2016. Indirect vector transmission is predominant at small distances, but transmission between distant herds and between countries usually occurs through movements of infected cattle or through vectors found mainly in animal trucks.

**Methods and principal findings:**

In order to estimate the threat for France due to the introduction of vectors found in animal trucks (cattle or horses) from at-risk countries (Balkans and neighbours), a quantitative import risk analysis (QIRA) model was developed according to the international standard. Using stochastic QIRA modelling and combining experimental/field data and expert opinion, the yearly risk of LSDV being introduced by stable flies (*Stomoxys calcitrans*), that travel in trucks transporting animals was between 6 x 10^−5^ and 5.93 x 10^−3^ with a median value of 89.9 x 10^−5^; it was mainly due to the risk related to insects entering farms in France from vehicles transporting cattle from the at-risk area. The risk related to the transport of cattle going to slaughterhouses or the transport of horses was much lower (between 2 x 10^−7^ and 3.73 x 10^−5^ and between 5 x 10^−10^ and 3.95 x 10^−8^ for cattle and horses, respectively). The disinsectisation of trucks transporting live animals was important to reduce this risk.

**Conclusion and significance:**

The development of a stochastic QIRA made it possible to quantify the risk of LSD being introduced in France through the import of vectors that travel in trucks transporting animals. This tool is of prime importance because the LSD situation in the Balkans is continuously changing. Indeed, this model can be updated to process new information on vectors and the changing health situation, in addition to new data from the TRAde Control and Expert System (TRACES, EU database). This model is easy to adapt to different countries and to other vectors and diseases.

## Introduction

The lumpy skin disease virus (LSDV) is a dsDNA virus belonging to the *Poxviridae* family and the *Capripoxvirus* genus [1]. Lumpy skin disease (LSD) is a highly contagious transboundary disease in cattle that produces major economic losses [2]. All ages and breeds of cattle are affected but calves and cows in the peak of lactation more severely so [3]. Clinical signs occur at the cutaneous (firm nodules of 0.5–5.0 cm in diameter occupying the entire thickness of the skin) and subcutaneous (oedema) levels. The nodules either regress or progress to necrosis, ulcers and finally scars. These lesions can also appear in other tissues such as the respiratory, digestive and genital tracts as well as in the lymph nodes [4].

The disease was first described in Zambia in 1929 and remains endemic to sub-Saharan Africa. The first outbreaks outside this zone appeared in Egypt in 1988 [5] and some months later in Israel in 1989 [6]. Since then, laboratory-confirmed foci have been observed in the Arabian Peninsula and in the Middle East (i.e. Turkey, Cyprus, Azerbaijan, Iran, Iraq, Israel, Jordan, Kuwait, Lebanon and the Palestinian Autonomous Territories). The disease was reported in Greece in August 2015. In 2016, it spread through different Balkan countries (i.e. Bulgaria, Former Yugoslav Republic of Macedonia, Serbia, Kosovo, Albania and Montenegro) [7].

As the epizootic spread, the affected European countries implemented policies of partial or total slaughter, disinsectisation and restriction of movement. Reactive vaccination measures have also been implemented, mainly in infected areas and in their periphery.

Although direct cattle-to-cattle transmission has been anecdotally reported, LSD is mainly transmitted by vectors [8,9]. The virus is probably transmitted by purely mechanical vectors [10]. Almost all blood-sucking arthropods (stable flies (*Stomoxys calcitrans*), horseflies (*Tabanidae*), mosquitoes and ticks) may play a potential role in the spread of LSDV between cattle. Indeed, *Stomoxys calcitrans* is a hematophagous fly widely distributed that can transmit sheep poxvirus and goat poxvirus [11]; it is also considered a major vector of LSDV [12]. Epidemiological data indicate that it plays a significant role in the dissemination of the virus within herds [12]. However, the experimental results are more ambiguous. In laboratory conditions, transmission was not evident since 200 *Stomoxys* fed 24 hours apart on viraemic cattle and then on healthy cattle did not transmit LSDV [13].

**Fig 1:**
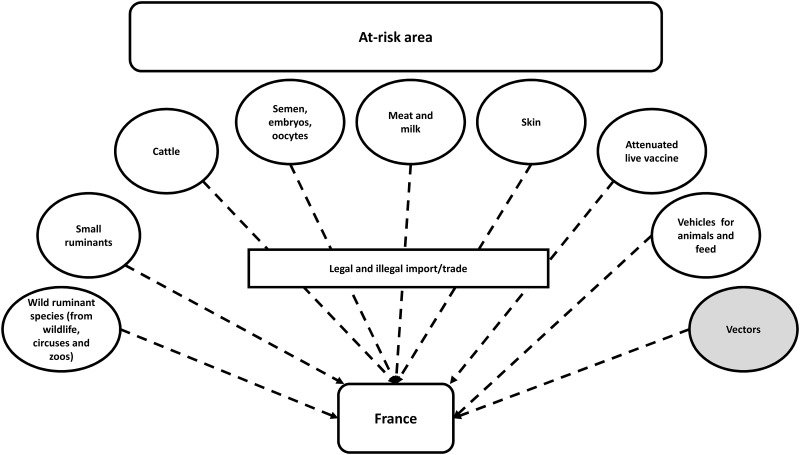
Risk pathways of LSD introduction from the at-risk area into France. In grey, the modality estimated in this study.

Transmission between distant herds and distant countries usually occurs through movements of infected cattle [14,15] but infected vectors can also introduce the virus into disease-free populations [16] (Fig 1). Vectors can move over short, medium and long distances in three different ways (i.e. transmitted by wind, trucks or birds).

Spontaneous movements of insects seem to be significant for the spread of the disease and only over short distances, due to the small range of dispersion of the vectors. For stable flies, [17] observed that less than 5% of the captured-tagged insects were recaptured more than 5 km from their place of release. These flies are also the main hypothesis for the first introduction of LSD in Israel, in August 1989, since *Stomoxys* spp. are the most common blood-borne insects in Israel and no animals had been introduced into the infected herds [18].

Recently, another species of fly, *Haematobia irritans*, was also suggested to be a potential LSD vector in Israel, but this proposal arose only from the concomitant observation of abundant populations of this fly and the first cases of LSD in beef cattle, without any formal evidence of its role [12]. There is currently very little information on the arthropod vectors involved in the spread of the LSDV in Eastern Europe.

Transport of vectors by truck seems to represent a risk of transmission over long distances [16]. Stable flies (*Stomoxys calcitrans*) can pose a risk during the transport of live animals, since they can remain on smooth surfaces for long periods if they are close to blood meal sources, i.e. mainly cattle and horses [10,19], even if the truck is open. On the contrary, the probability of introduction by other vehicles (transport of food, passenger cars, etc.) is negligible since these flies are always close to cattle or equines. Other vector species probably play only a minor role: *Tabanidae* live outdoors [14] and do not enter buildings or vehicles. If a specimen of this species enters a truck, it rapidly wrecks its wings, loses its flying ability and dies within only a few hours (P. Jacquiet, personal communication), making its role in long-distance spread unlikely.

*Aedes aegypti* can transmit LSDV for up to six days after infection [20], but this species is not present in Europe [21] and the similar species *Aedes albopictus* is essentially anthropophilic [22], which means it is very unlikely to be transported by a cattle truck and bite cattle.

Transmission by vector-carrying birds may also be possible; this kind of transport has been described only for ticks [23–25]. Although the presence of LSDV in African ticks was documented in the field conditions [26], the trans-stadial and trans-ovarian transmission was documented only in experimental conditions [27–30]. However, the role of European tick species currently remains unknown and merits attention.

With more than 3,000 outbreaks of LSD registered from Balkan countries during the four late years in the Animal Disease Notification System (ADNS) of the European Commission, the estimation of the risk of introduction of Lumpy Skin Disease in France by the import of vectors in animal trucks is useful. Beside, quantitative import risk assessment (QIRA) has been widely used to provide scientific evidence for policy decisions relating to different diseases at both national and international levels [31–32]. Moreover, OIE international standard exists to conduct QIRA modelling [33].

The objective of this study was to quantify the annual probability of a first outbreak of LSD occurring in France due to the introduction of vectors that travel in trucks transporting live animals (cattle and horses) and using QIRA modelling.

## Material and methods

### At-risk area

For the purpose of this study, the at-risk area was defined as the Balkan disease free regions of affected/infected countries on 1 January 2017 (Greece, Bulgaria, Former Yugoslav Republic of Macedonia, Serbia, Kosovo, Albania and Montenegro) as well as their non-infected neighbouring countries (Romania, Croatia, Hungary, Ukraine, Bosnia and Herzegovina).

### Routes of vector introduction

It has been assumed that vectors can move over long distances in three different ways: transported by vehicles, by themselves through wind, and by birds. For movements of vehicles, only trucks transporting live animals (mainly cattle and horses due to host preference) [10,34] have been considered since the probability for other vehicles seems very low. For movements of vectors through cattle transport, disease free regions of affected/infected countries as well as their non-infected neighbouring countries have been considered. Since horses are as attractive as cattle to these vectors [10,34], the transport of horses needs to be taken into account as well. Given the distance between the current at-risk area and France, a truck would need to travel for two to three days.

### Probabilities considered in the model

An expert opinion was organised with six selected international LSD specialists, mostly from newly infected countries. After these specialists were first contacted by e-mail, a short explanation of the hypotheses and arguments used in the model was sent to the experts. This information was attached to a questionnaire with a first proposal of values for the input parameters included in the model so they would correct them or review their reliability. The responses were then discussed by the authors and used to choose the final values for each probability.

**Fig 2:**
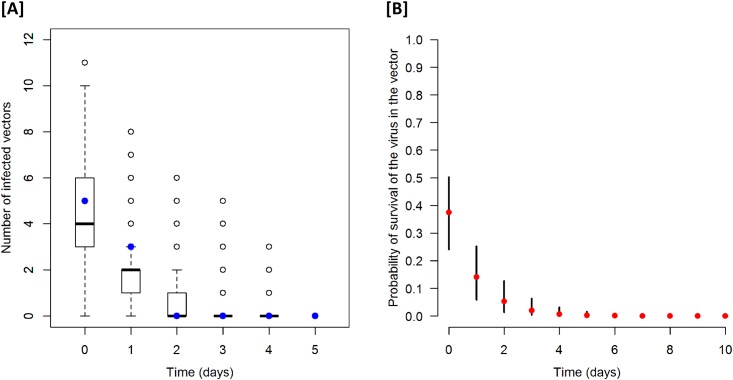
Reported and posterior predicted numbers of infected vectors [A] and Posterior probability of LSDV surviving in *Stomoxys calcitrans* [B]. [A] The blue dots refer to the experimental results reported by [13], and the boxplots refer to the distribution of model predictions using the estimated posterior distribution; [B] Time refers to the number of days after an infective blood meal. The solid black lines and the red dots show the 95% credible interval and the median of the posterior distribution.

The model includes ten probabilities as input parameters (Table 1):

**P1**—Probability of importing cattle from an at-risk area (i.e. disease-free regions of an affected country or from a non-affected neighbour country) that can become infected with LSDV before its detection.
  The incidence of countries newly affected by LSD in 2016 was established at 0.07 per month based on the progress of the disease in 2016 in the Balkan countries (seven affected countries per 100 at-risk months).
  The high-risk period (time between the first animal becoming infectious and the diagnosis/reporting of the disease) was estimated at one month. At the beginning of infection, the first lesions are mild and usually remain unobserved by farmers, especially in areas where the disease has not been previously diagnosed.
**P2**—Probability of importing animals from an infected farm located in the zone defined in P1. It was assumed that in a recently infected region, the percentage of infected farms would be very low.
**P3**—Probability of a *Stomoxys* being infected. We assumed that the proportion of infected *Stomoxys* should be equivalent to the proportion of contagious cattle, which means it should be the same as the probability of infection for a given animal from an infected farm defined in P2. The median intra-herd prevalence of clinical cases observed in Greece and Bulgaria between May and August 2016 was 3% with a minimum of 0.3% and a maximum of 25%. According to [1], only 50% of infected animals show clinical signs. We have assumed that diseased animals will not be sold.
**P4**—Probability of the virus surviving in the vector. There is little information about the survival of LSDV in insects. Chihota et al. [13] were able to detect LSDV by PCR in *Stomoxys calcitrans* feeding on a LSDV-infected cattle. Five out of 12 vectors were PCR-positive on the day of the blood meal, three were positive on the next day, and none were positive on the following days.
  Based on these experimental data and the hypothesis that all vectors had an infectious blood meal at the start of the experiment (on day 1), the probability *p*_*d*_ of a vector still being infected (i.e. PCR-positive) on day *d* was:
  $$p_{d}={\left(1-r\right)}^{d}$$ (1)
  Where *r* is the daily probability of recovery (i.e. vectors becoming PCR-negative). Using a Bayesian framework, the posterior probability of recovery *r* was estimated using a uniform distribution *U* (0,1) as prior and a likelihood function expressed as a binomial process:
  $$L=\prod_{d}\left(\begin{array}{c} n_{d}\\ k_{d} \end{array}\right)p_{d}{}^{k_{d}}{\left(1-p_{d}\right)}^{n_{d}-k_{d}}$$ (2)
  Where *n*_*d*_ is the number of vectors tested on day *d* by [13] and *k*_*d*_ is the number of PCR-positive vectors on day *d*. The median of the posterior probability of recovery was estimated to be 0.626 (95% credible interval: 0.497–0.759) (Fig 2A and 2B).
**P5**—Probability of *Stomoxys* surviving during transport. The lifespan of *Stomoxys* fed with blood from cattle or horses is documented in experimental conditions [35]. The mean time required to achieve natural mortality of 100% is about 15 days, which means that daily mortality is about 7% (i.e. 1/15). We have considered that the transport between the current at-risk area and France takes between two and three days.
**P6**—Probability that LSDV is transmitted to the destination cattle, in the event of a truck transporting cattle to a farm. There is a lack of knowledge about the number of *Stomoxys* bites required to transmit the virus. A worst-case scenario has been considered.
**P7**—Probability that LSDV is transmitted at the destination in the event of a truck transporting cattle to a slaughterhouse. In this case, it is most likely that the *Stomoxys* arriving with the truck will bite cattle to be slaughtered and therefore infection will not spread. However, if there is a cattle farm in the immediate vicinity, some stable flies can move to this farm and infect animals. The probability was fixed according to an expert opinion.
**P8**—Probability that LSDV is transmitted to cattle at the destination in the event of a truck transporting horses. Horses transported between countries are mostly used for leisure, and there are usually no cattle near the origin and destination sites. Moreover, horses are as attractive as cattle to *S*. *calcitrans*. In the event of a mixed farm, we can assume that the insects that live near horses and near cows belong to different subpopulations [10,34]. The probability was fixed according to an expert opinion.
**P9 and P10**—Probability that horses come from a mixed farm (containing cattle) or that a cattle farm is in the immediate vicinity of the stables (P9), and probability that horses go to a mixed farm or that there is a cattle farm in the neighbourhood (P10). Of 34,500 horse facilities recorded in France in 2013, a total of 3,420 had mixed cattle and equine activities [36]. No information was available for the country of origin of horses (at-risk area). For this reason, the value of P9 was assumed to be the same as that of P10.

**Table 1:** Summary of the input variables used to calculate the risk of LSDV being introduced in France through *Stomoxys* travelling inside trucks transporting live animals (cattle and horses).

Input variable	Data source	Min	Mode	Max	Variable	Distribution
Probability of importing cattle from an at-risk area that can become infected with LSDV before its detection	Combination of experimental data and expert opinion	0.05	0.07	0.2	P1	RiskPert(0,05;0,07;0,2)
Probability that trucks come from an infected farm located in the at-risk area	Expert opinion	0.005	0.0075	0.01	P2	RiskPert(0,005;0,0075;0,01)
Probability of a animal being infected without clinical signs in the farm	Field data and expert opinion The proportion of infected *Stomoxys* is assumed to be the same as the proportion of contagious cattle	0.003	0.03	0.25	P3	RiskPert(0,003;0,03;0,25)
Probability of the virus surviving in the *Stomoxys*	Combination of experimental data, expert opinion and Bayesian modelling	0.0197 (3 days after the infective blood meal)	-	0.0525 (2 days fter the infective blood meal)	P4	RiskUniform(0,0197;0,0525)
Probability of *Stomoxys* surviving during transport (2–3 days)	Combination of experimental data and expert opinion	0.8	-	0.9	P5	RiskUniform(0,8;0,9)
Probability that LSDV is transmitted at the destination in the event of a truck transporting cattle to a farm	Worst-case scenario	-	1	-	P6	
Probability that LSDV is transmitted at the destination in the event of a truck transporting cattle to a slaughterhouse*	Expert opinion	0.001	-	0.01	P7	RiskUniform(0,001;0,01)
Probability that LSDV is transmitted at the destination in the event of a truck transporting horses	Expert opinion	0.001	-	0.01	P8	RiskUniform(0,001;0,01)
Probability that horses come from a mixed farm (with cattle) or that a cattle farm is in the vicinity of the stables	Interbev [36]	0	0.099	1	P9	RiskBeta(0;0,099;1)
Probability that horses go to a mixed farm (with cattle) or that a cattle farm is in the vicinity of the stables	Interbev [36]	0	0.099	1	P10	RiskBeta(0;0,099;1)
Number of *Stomoxys* per cattle	Combination of experimental data and expert opinion	0.1	3.9	22.22	sb	RiskPert(0,1;3,9;22,22)
Number of *Stomoxys* per horse	Combination of experimental data and expert opinion	0.2	3.2	6	sh	RiskPert(0,2;3,2;6)
Number of batches of cattle transported yearly to cattle farms	TRACES	3	7	11	n1	RiskPert(3;7;11)
Number of batches of cattle transported yearly to slaughterhouses	Scenario	3	7	11	n2	RiskPert(3;7;11)
Number of batches of horses transported yearly	TRACES	22	44	66	n3	RiskPert(22;44;66)
Number of cattle transported yearly to cattle farms	TRACES	90	182	270	a1	RiskPert(90;182;270)
Number of cattle transported yearly to slaughterhouses	Scenario	90	182	270	a2	RiskPert(90;182;270)
Number of horses transported yearly	TRACES	22	44	66	a3	RiskPert(22;44;66)
Number of *Stomoxys* per cattle transported x Number of animals in the truck travelling to a cattle farm	Calculation	3	101.4	545.4	N1f	sb x (a1 / n1)
Number of *Stomoxys* per cattle transported x Number of animals in the truck travelling to a cattle slaughterhouse	Calculation	3	101.4	545.4	N1a	sb x (a2 / n2)
Number of *Stomoxys* per horse transported x Number of horses in the truck	Calculation	0.2	3.2	6	N1h	Sh x (a3 / n3)

Prob: Probability; Min: Minimum; Max: Maximum;

*: No batches of animals for slaughterhouse coming from the at-risk area. In order to evaluate the potential impact of introducing batches of animals to be slaughtered, a scenario was developed using the same number of batches of cattle introduced on farms.

### Number of insects

The number of insects transported by truck was estimated based on the literature. A key aspect of the risk of viral introduction is the number of *Stomoxys* that can be transported by truck from the at-risk area. The adults of both sexes are haematophagous [10]. We assumed that all flies in the truck are feeding or have fed on the transported cattle or horses because they have to be nourish during so long period and they have a main attraction to these species [10,19]. The number of *S*. *calcitrans* seen resting or feeding on dairy cattle and horses was counted once a week for one year [37]. These counts increased in the spring, stabilised during the summer and early autumn, and decreased from the autumn until the following spring.

For dairy cattle, they were as follows: minimum of 0.1, maximum of 9.3, and median of 3.9. In addition, based on the counts of stable flies per front leg and the fact that nearly 45% of the total feeding flies were on the front legs [38], the number of stable flies per cattle was estimated in some other studies [39–41]. Because these studies were performed in the period of peak stable-fly activity, the average maximum count of stable flies per cattle was extended to 22.22. For horses [37], the counts were as follows: minimum of 0.2, maximum of 6, and median of 3.2. We assumed that the number of stable flies that travel in trucks transporting animals is proportional to the number of animals in the batch.

### Cattle and horse movements

The number of cattle and horse movements was calculated based on the TRAde Control and Expert System (TRACES, EU database).

According to TRACES, during the one-year period from July 2015 to July 2016, only 182 cattle originating from the at-risk area were introduced in France through seven batches coming from Romania and Hungary (Table 1). During this period, no animals were imported from at-risk countries outside the EU. All the batches of animals were introduced on French farms (= **n1**). No animals came from at-risk countries to be slaughtered. In order to evaluate the potential impact of vectors transported by trucks going to slaughterhouses (= **n2**), a scenario was developed using the same number of batches of cattle introduced on farms.

Between September 2015 and September 2016, 44 horses were introduced into French facilities for breeding, from Bulgaria, Croatia, Greece and Hungary. All movements involved a single animal (data from TRACES). The number of batches of horses considered was 44 (= **n3**).

### Calculation of the probability of the LSDV being introduced in France through vectors transported by trucks

**Fig 3:**
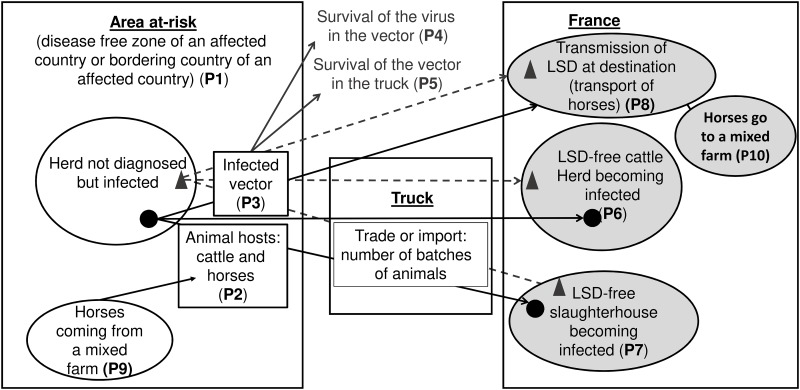
Conceptual framework for the estimation of the risk of lumpy skin disease being imported into France through vectors. P1 to P10 are probabilities defined in the section named “Probabilities considered in the model”; Circular markers: animals; Triangular markers: vectors; Solid lines: transmission route for animals; Dotted lines: transmission route for vector; Blank circles: herd/farm in the area at-risk; Shaded circle: herd/farm in France.

The conceptual framework for the estimation of the risk of LSD being introduced in France through vectors transported by trucks is depicted in Fig 3.

The model considers three worst-case scenarios, which consist in:

- (i) the absence of cleaning, disinfection and disinsectisation of the truck used for the transport of animals, even though these procedures are required according to the European legislation [42],
- (ii) the absence of unloading of animals during transport, even though this procedure is required for movements exceeding eight hours [43] and
- (iii) the probability of infecting cattle on the destination farm is 100%.

The input parameters above (Table 1) were used to calculate the probability of the LSDV being introduced in France.

We calculated the probabilities of an LSD outbreak occurring in France through vectors transported by trucks.

**R1**—Probability that an infected *Stomoxys* reaches its destination, with:
  Cattle for farms:
  $$R1_{f}=1-{\left(P1*P2*P3*P4*P5\right)}_{}^{N1f}$$ (3)
  Cattle for slaughterhouses:
  $$R1_{a}=1-{\left(P1*P2*P3*P4*P5\right)}^{N1a}$$ (4)
  Horses:
  $$R1_{h}=1-{\left(P1*P2*P3*P4*P5\right)}^{N1h}$$ (5)
**R2, R3 and R4**—Probability that a native cattle becomes infected with *Stomoxys* that travelled with cattle going to farms (R2) or going to slaughterhouses (R3) or that travelled with horses (R4):
  $$R2=1-{\left(1-R1_{f}*P8\right)}^{n1}$$ (6)
  $$R3=1-{\left(1-R1_{a}*P9\right)}^{n2}$$ (7)
  $$R4=1-{\left(1-R1_{h}*P10*P11*P12\right)}^{n3}$$ (8)

### Quantitative import risk assessment modelling

The spreadsheet model was designed in Microsoft Excel 2013 (Microsoft, Redmond, WA). The model was run for 100,000 iterations using Monte Carlo sampling in @Risk version 7.5 (Palisade Corporation, Ithaca, NY).

A Pert distribution was used for variables with a modal value for which minimum and maximum values were known. Beta distributions were used for the probability of mixed farms (horses and cattle) and uniform distributions were used when the expected value had the same probability for each point between the minimum and maximum values.

### Sensitivity analysis

A sensitivity analysis was performed in order to identify the inputs that had a greater influence on the final outputs. This sensitivity analysis was carried out using the rank order correlation method, based on the Spearman rank correlation coefficient calculations. The rank correlation coefficient is calculated between the selected output variable and the samples for each of the input distributions. The higher the correlation between the input and the output, the more significant the input is in determining the value of the output.

### Ethic statement

The ethic statement is not required because they are no specific experimentation, no specific epidemiological survey for the QIRA model developed. This QIRA model is based only on reasoning, literature review, expert opinion and development of mathematical model.

## Results

The Table 1 shows the main characteristics of the probabilities introduced in the model.

The probability of the LSDV being introduced in France through vectors transported by trucks using a quantitative import risk assessment modelling was calculated.

The yearly risk of LSDV being introduced by insects that travel in trucks transporting animals is between 6 x 10^−5^ and 5.93 x 10^−3^ (95% confidence interval) with a median value of 89.9 x 10^−5^; it is mainly due to the risk related to the transport of cattle from at-risk countries that enter farms located in France. The risk related to the transport of cattle going to slaughterhouses or the transport of horses is significantly lower (with confidence intervals between 2 x 10^−7^ and 3.73 x 10^−5^ and between 5 x 10^−10^ and 3.95 x 10^−8^ for cattle and horses respectively) (Table 2).

**Table 2:** Probabilities of LSDV being introduced in France by *Stomoxys* travelling with cattle or horses transported from the Balkan countries.

Probability	2.5 Percentile	Median	97.5 Percentile
**R1**—Probability that an infected *Stomoxys* reaches its destination			
R1_f_ (cattle for farms)	1 x 10^−5^	13.10 x 10^−5^	93 x 10^−5^
R1_a_ (cattle for slaughterhouses)	1 x 10^−5^	13.10 x 10^−5^	93 x 10^−5^
R1_h_ (horses)	0.3 x 10^−6^	2.88 x 10^−6^	15.3 x 10^−6^
**R2**—Probability that a native cattle becomes infected with *Stomoxys* that have travelled with cattle going to farms	6 x 10^−5^	89.9 x 10^−5^	593 x 10^−5^
**R3**—Probability that a native cattle becomes infected with *Stomoxys* that have travelled with cattle going to slaughterhouses (scenario)	0.2 x 10^−6^	4.27 x 10^−6^	37.3 x 10^−6^
**R4**—Probability that a native cattle becomes infected with *Stomoxys* that have travelled with horses	0.5 x 10^−9^	5.82 x 10^−9^	39.5 x 10^−9^

### Sensitivity analysis

According to the sensitivity analysis, the four parameters that had a greater effect on the final model outputs were the probability of trucks coming from the at-risk area (P1), the probability of *Stomoxys* being infected (P3), the probability of the virus surviving in the vector (P4), and the number of *Stomoxys* in the truck (sb or sh). For the probability that a native cattle becomes infected with *Stomoxys* that have travelled with cattle going to slaughterhouses (R3) or with horses (R4), additional influential parameters were the probabilities that LSDV is transmitted if the truck transports cattle to a slaughterhouse (P7) or transports horses (P8).

**Fig 4:**
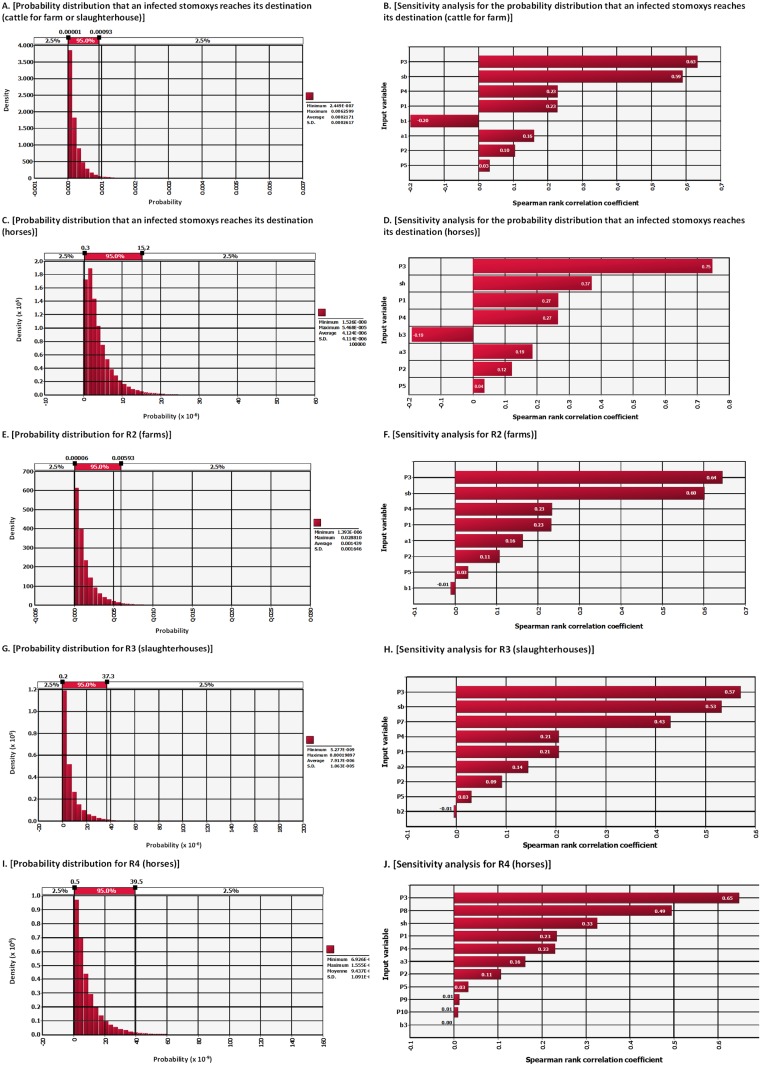
Distribution of probabilities that vectors inside trucks of animals (cattle or horses) transmit LSD to native animals and sensitivity analysis. A, C, E, G, and I represent the probabilities; B, D, F, H and J represent the sensitivity analyses (Spearman rank correlation coefficients are presented in decreasing order of importance).

The distribution of the calculated probabilities are shown in Fig 4A, 4C, 4E, 4G and 4I and the results of the sensitivity analysis are shown in Fig 4B, 4D, 4F, 4H and 4J.

## Discussion

Using a QIRA modelling, the probability of an LSD outbreak occurring in France following the introduction of vectors by trucks that transport cattle was estimated at between 6 x 10^−5^ and 5.93 x 10^−3^ with a median value of 89.9 x 10^−5^.

According to these results, when we evaluate the risk of LSD being introduced through the transport of cattle, we have to take into account not only the risk posed by the animals, but also the possible risk of transmission by the insects—especially the stable flies—that travel with them. The risk due to vectors can be easily reduced by disinsectising the vehicles. In fact, the European legislation requires the cleaning, disinfection and disinsectisation of vehicles before leaving an at-risk area [42]. It is not certain that the quality of vehicle disinsectisation (e.g. by fumigation) is strictly controlled. For this reason, we have considered the worst-case scenario in the model, where trucks are never disinfected and disinsectised; according to these results, efforts need to be made to apply the legislation in order to reduce the risk of LSD being introduced in disease-free areas.

The journey between the Balkan countries and France lasts between two and three days. During this period, animals have to be unloaded for several hours for a rest according to the European legislation on animal welfare [43]. If the animals leave the truck, *Stomoxys* will follow them and exit. There is also the possibility they will go to another truck or stay in the centre of assembly if other animals are present. Indeed, the unloading of animals (and associated infected vectors) during travel can also introduce LSD infection in countries other than France. The maximum permitted duration for the road transport of adult cattle is currently eight hours for undeveloped vehicles (i.e. unloading of animals needed) and 29 hours for developed vehicles (i.e. unloading of animals not needed) [44–45]. It should be noted that the provisions relating to outbreaks seem to be called into question because of the risk of infectious diseases spreading between animals of different origins and the extra stress caused by their unloading and reloading compared to the ability to rest in the vehicle under conditions that meet the regulatory requirements [45]. For these reasons, the QIRA model considers only the scenario that corresponds to the non-unloading of animals.

There are currently no cattle introduced for slaughter in France from the at-risk area, and therefore the current risk cannot be estimated. If cattle came to be slaughtered in France (scenario), the risk would be much lower than the risk for animals sent to farms. The risk related to vectors that travel with horses is still lower than the risk due to the transport of animals to be slaughtered.

During the period in which we obtained the data on animal movements, no horses were introduced from zones where LSD is present. Considering that in the event of an LSD outbreak, horse movements are not banned, it is important to disinsectise trucks coming from affected zones in order to minimise the risk.

According to the sensitivity analysis, some interesting mitigating measures were highlighted, such as the importance of early LSD detection in the country of origin (limiting the spread of LSD), the importance of vaccine campaigns in LSD-affected countries (reducing the circulation of LSDV), and the importance of reducing the number of *Stomoxys* in the truck through its cleaning, disinfection and disinsectisation according to the European legislation [42]. These measures should be promoted to limit the risk of importing vectors that travel inside trucks with animals.

In addition, it should be noted that the long-distance transport of animals intended for slaughter should be replaced, as far as possible, with the transport of carcasses.

In the QIRA model, the regional variability of mixed horse/cattle farms has not been taken into account due to the lack of information. Indeed, available data from France were used as surrogate data to simulate the situation in the country of origin of travelling animals [36].

Regarding other routes of vector introduction, the risk associated with the transport of vectors by birds is considered negligible for two reasons. Firstly, it is unlikely that a tick transported by a bird will take a blood meal on cattle. Secondly, the current LSD-infected area and France are not located along the main migration flyways for birds. For these reasons, the probability of LSD carried by birds being introduced into France is estimated to be almost null. However, there is little knowledge about the role played by European ticks in the transmission of the LSDV.

Transport by wind has been mentioned for the spread of LSDV in Eastern Europe and the Middle East [46]. However, considering the distance between the currently infected area and France, and the prevailing winds in Europe, the probability that LSDV carriers are passively transported by winds and transmit the disease in France is negligible.

There are some limitations of the present QIRA modelling related to the choice of assumptions and worst case scenarios (proportion of infected *Stomoxys* equivalent to the proportion of contagious cattle, absence of cleaning, disinfection and disinsectisation of the truck used for the transport of animals, absence of unloading of animals during transport, only *Stomoxys calcitrans* considered as mechanical vector of LSDV, proportion of mixed cattle and equine activities in countries of origin unknown and consequently estimated at the same as in France, and probability of infecting cattle on the destination farm of 100%).

Concerning the LSD and LSDV, some data needs are related to the time between infection of a farm and the detection of the disease, the actual duration of the transport and the number of unloads, the survival of the LSDV in the crop of *Stomoxys*, the effectiveness of transmission by bites (number of bites needed), the effective insect eradication in animal transport trucks (insecticide treatment or alternative control methods), the density/dynamic of *Stomoxys calcitrans* in the European farms and the risks associated with other European blood-sucking insects.

In conclusion, the development of a stochastic QIRA modelling made it possible to quantify the risk of LSD being introduced in France through the import of vectors that travel in trucks transporting animals. This estimation was limited to the risk of introduction by *Stomoxys calcitrans*. The disinsectisation of trucks transporting live animals is of prime importance to reduce this risk.
